# Dynamic response of a long cylinder under thermal shock in micropolar thermoelasticity

**DOI:** 10.1371/journal.pone.0293849

**Published:** 2023-12-20

**Authors:** Hany H. Sherief, Mohamed F. Abbas, Mohamed F. Zaky, Samar A. Mahrous

**Affiliations:** 1 Faculty of Science, Department of Mathematics, Alexandria University, Alexandria, Egypt; 2 Institute of Basic and Applied Science, College of Engineering, Arab Academy for Science, Technology and Maritime Transport, Alexandria, Egypt; The British University in Egypt, EGYPT

## Abstract

In this manuscript, the dynamic response of a long cylinder subjected to an asymmetric thermal shock is investigated within the framework of generalized micropolar thermoelasticity. The displacement and micro-rotation are assumed to vanish at the surface. Laplace transformation techniques are used to solve the problem. The solution is obtained in the transformed field using an innovative direct approach. Furthermore, we obtain the inverse transformations using a numerical method based on Fourier expansion. The obtained results are carefully presented through graphical representations and discussed extensively across different relaxation time values. It is evident that the relaxation time parameter significantly influences all the distributions. The displacement distributions are always continuous, whereas all other functions, including temperature variation, stress distribution, and micro-rotation, exhibit discontinuity at the wave front. The results obtained hold significant importance in various technological applications and in the manufacturing of mechanical components.

## 1. Introduction

Thermoelasticity theory deals with the constitutive relationship among the mechanical and thermal fields of an elastic body. In the uncoupled thermoelasticity theory, the temperature is obtained from the heat equation where the effect of the mechanical state is ignored. In addition, the heat propagation speed based on this theory is infinite which contradicts the physical reality. M. Biot in 1956 [1] solved one of the problems of the uncoupled theory by introducing the coupled theory of thermoelasticity, which implies that any thermal variation in the medium leads to the presence of strain in the elastic body and vice versa. However, another problem with this theory remains unsolved which is the infinite heat propagation speed.

Different models have been postulated by different authors in the thermoelasticity field to solve this problem and obtain finite velocities of heat waves. In 1967, Lord and Shulman [2] introduced the generalized thermoelasticity theory with a single relaxation time for a particular case which is an isotropic media. In 1980, Lord and Shulman’s work was further extended by Sherief [3] and by Dhaliwal and Sherief [4] in order to cover an anisotropic media. In generalized thermoelasticity theory, the conventional Fourier law has been substituted with the Maxwell-Cattaneo law, which incorporates both heat flux and its temporal derivative. Therefore, the paradox of the infinite velocity of heat diffusion that characterizes the uncoupled and the coupled thermoelasticity theories was eliminated and the heat equation in the theory of generalized thermoelasticity is hyperbolic. Green and Lindsay [5], in 1972, developed what is called the theory of thermoelasticity with two relaxation times by generalizing a known thermodynamic inequality. This theory is also called the temperature-rate dependent theory. This theory also predicts finite speeds of wave propagation. Ignaczak [6, 7] examined the generalized thermoelasticity theory and proved its uniqueness. The stability of this theory has been studied by Sherief in 1987 who proved also the uniqueness of the theory [8]. Some works in the context of these two theories and their extensions can be found in [9–20].

In 1993, Green and Naghdi extended the theory of thermoelasticity and postulated new theories for homogeneous materials, called GN model I, II and III [21–23]. A good review of studies that used the generalized thermoelasticity theory can be found in [24]. The traditional theory of elasticity has been developed several times and gives agreeable outcomes in various engineering subjects with different structural materials. However, for materials with defined internal structure, the traditional theory of elasticity did not succeed in giving good outcomes [25].

Voigt generalized the traditional theory of elasticity which is symmetric to a non-symmetric theory of elasticity in order to improve the outcomes of the classical theory which led to the presence of couple stress in elasticity [26]. Cosserat brothers extended Voigt ‘s work and suggested that each particle of the material is capable of rotation in addition to the translation supposed in the classical theory of elasticity [27]. Then, Eringen extended Cosserat theory and formulated the micropolar theory of elasticity [28] which include the microinertia impacts.

Nowacki [29], Eringen [30] and Iesan [31] included the thermal properties into the micropolar theory and created the micropolar thermoelasticity theory. Micropolar thermoelasticity theory was studied by Tauchert et al. [32] who developed the basic mathematical equations of the micropolar thermoelasticity. Many researches have been done on the theory of micropolar thermoelasticity. Sherief and colleagues [33] formulated the theory of generalized micropolar thermoelasticity, which predicts a finite velocity of propagation for both thermal and mechanical impacts. Various contributions to this field of research are documented in the cited references [34–38].

In the present manuscript, we solve a two-dimensional problem within the framework of generalized micropolar thermoelasticity theory. The field variables, including temperature changes, displacement distributions, stress variations, and microrotation distributions, are obtained in an infinite cylinder with its surface subjected to asymmetric thermal shock. The displacement and micro-rotation are assumed to vanish on the surface. Laplace transformation methods are employed for solving the problem. In the transformed domain, the solution is obtained through the utilization of an innovative direct approach. The inverse transformations are computed using a numerical technique depend on Fourier expansion. To the best of our knowledge, no previous studies were found that specifically use this innovative approach without relying on potential functions. Potential functions have certain limitations. Firstly, they may not always be accessible. Secondly, on occasion, potential function solutions diverge, whereas solutions in terms of physical variables consistently converge. The obtained results are carefully presented through graphical representations and discussed extensively across different relaxation time values. It is evident that the relaxation time parameter significantly influences all the distributions. The results obtained hold significant importance in various technological applications and in the manufacturing of mechanical components.

## 2. Problem formulation

**Fig 1:**
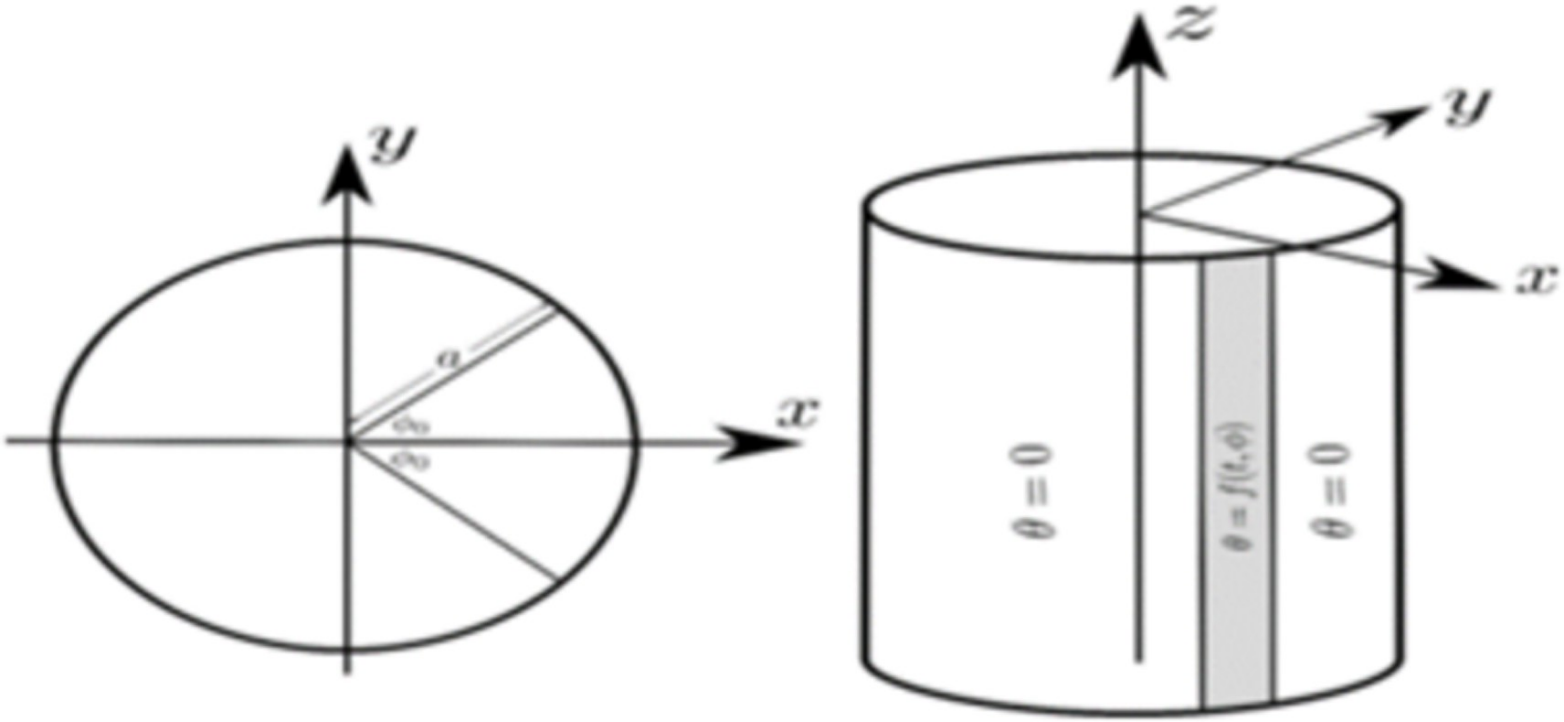
Schematic of the cylinder.

We shall consider an infinite cylinder of radius *a* formed of a micropolar homogeneous thermoelastic substance (as shown in Fig 1). We shall use the cylindrical coordinate variables {(*r*, *φ*, *z*): 0 ≤ *r* ≤ *a*, 0 ≤ *φ* ≤ 2*π*,—*∞* < *z* < *∞*} The surface of the cylinder is acted on by a thermal shock that is a function of the time *t* and the angle *φ*. The displacement and rotation components vanish on the surface. Thus, from the physics of the problem, all the considered fields will vary with *t*, *r* and *φ* and independent of *z*.

The components of the displacement and micro-rotation vectors will be in the following forms:

$$\underline{u}=\left(u_{r},\;u_{\varphi },\;0\right),\;\underline{\omega }=\left(0,\;0,\;\omega_{z}\right).$$

The cubical dilatation *e* is given by

$$e=\nabla \;.\;\overset{-}{u}=\frac{1}{r}\left(\frac{\partial }{\partial r}\left(ru_{r}\right)+\frac{\partial u_{\varphi }}{\partial \varphi }\right).$$ (1)

In the absence of body couples, body forces and heat sources, the governing equations will be [33]:

$$\left(\mu +\lambda -\alpha_{p}\right)\nabla [\nabla \;.\;\underline{u}]+\left(\alpha_{p}+\mu \right)\nabla^{2}\underline{u}+2\alpha_{p}\nabla \;\times \;\underline{\omega }-\gamma \nabla \theta =\rho \frac{\partial^{2}\underline{u}}{\partial t^{2}},$$ (2)

$$2\alpha_{p}\nabla \;\times \;\underline{u}-4\alpha_{p}\underline{\omega }+\left(v_{p}+\varepsilon_{p}-\beta_{p}\right)\nabla [\nabla \;.\;\underline{\omega }]+\left(\beta_{p}+v_{p}\right)\nabla^{2}\underline{\omega }=\rho J\frac{\partial^{2}\underline{\omega }}{\partial t^{2}},$$ (3)

$$\frac{\partial }{\partial t}\left(1+\tau_{0}\frac{\partial }{\partial t}\right)\left(c_{E}\rho \theta +T_{0}\gamma e\right)=k\nabla^{2}\theta ,$$ (4)

where *μ*, *λ* are material constants equivalent to Lamé’s constants for the generalized thermoelasticity theory. *ρ* is the mass density, *γ* = (3*λ* + 2*μ*)*α*_*t*_, where *α*_*t*_ is the linear thermal expansion coefficient, *T* is the absolute temperature, *T*_0_ is a reference temperature satisfying |(*T*–*T*_0_)/*T*_0_| << 1 and *θ* = *T–T*_0_. *τ*_0_ is the relaxation time, *k* is the thermal conductivity, *c*_*E*_ is the specific heat at constant strain, *J* is the non-vanishing component of the micro-rotation tensor while *α*_*p*_, *ε*_*p*_, *β*_*p*_ *and υ*_*p*_ are new constants of micropolar thermoelasticity (see [33]).

The operator ∇^2^ in our case is given by

$$\nabla^{2}=\frac{\partial^{2}}{\partial r^{2}}+\frac{1}{r}\;\frac{\partial }{\partial r}+\frac{1}{r^{2}}\;\frac{\partial^{2}}{\partial \varphi^{2}}$$ (5)

The non-zero components *σ*_*ij*_ of the stress tensor are [33]:

$$\sigma_{rr}=(\lambda +2\mu )\frac{\partial u_{r}}{\partial r}+\lambda \left(\frac{1}{r}\;\frac{\partial u_{\varphi }}{\partial \varphi }+\frac{u_{r}}{r}\right)-\gamma \theta$$ (6)

$$\sigma_{zz}=\lambda \left(\frac{\partial u_{r}}{\partial r}+\frac{1}{r}\;\frac{\partial u_{\varphi }}{\partial \varphi }+\frac{u_{r}}{r}\right)-\gamma \theta$$ (7)

$$\sigma_{\varphi \varphi }=(\lambda +2\mu )\left(\frac{1}{r}\;\frac{\partial u_{\varphi }}{\partial \varphi }+\frac{u_{r}}{r}\right)+\lambda \left(\frac{\partial u_{r}}{\partial r}+\frac{u_{\varphi }}{r}\right)-\gamma \theta$$ (8)

$$\sigma_{\varphi r}=\left(\mu -\alpha_{p}\right)\;\frac{\partial u_{\varphi }}{\partial r}+\left(\mu +\alpha_{p}\right)\left(\frac{1}{r}\;\frac{\partial u_{r}}{\partial \varphi }-\frac{u_{\varphi }}{r}\right)+2\alpha_{p}\omega_{z}$$ (9)

$$\sigma_{r\varphi }=\left(\mu +\alpha_{p}\right)\frac{\partial u_{\varphi }}{\partial r}+\left(\mu -\alpha_{p}\right)\left(\frac{1}{r}\;\frac{\partial u_{r}}{\partial \varphi }-\frac{u_{\varphi }}{r}\right)+2\alpha_{p}\omega_{z}$$ (10)

The non-zero components *μ*_*ij*_ of the couple stresses are [33]:

$$\mu_{rz}=\left(\gamma +\varepsilon_{p}\right)\frac{\partial \omega_{z}}{\partial r}$$ (11)

$$\mu_{zr}=\left(\gamma -\varepsilon_{p}\right)\frac{\partial \omega_{z}}{\partial r}$$ (12)

$$\mu_{\varphi z}=\frac{1}{r}\left(\gamma +\varepsilon_{p}\right)\frac{\partial \omega_{z}}{\partial \varphi }$$ (13)

$$\mu_{z\varphi }=\frac{1}{r}\left(\gamma -\varepsilon_{p}\right)\frac{\partial \omega_{z}}{\partial \varphi }.$$ (14)

We assume that the initial conditions are homogeneous. The boundary conditions are as follows:

θ=f(t,φ)onr=a 15a

$$u_{r}=u_{\varphi }=w_{z}=0\;\text{on}\;r=a$$ (15b)

Where *F*(*t*, *φ*) is a known function

We can express the governing equations in a more convenient form by employing the following dimensionless quantities

$$\left(r^{\prime },\;u_{r}^{\prime },\;u_{\varphi }^{\prime }\right)=v\eta \left(r,\;u_{r},\;u_{\varphi }\right),\;t^{\prime }=v^{2}\eta t,\;\tau_{0}^{\prime }=v^{2}\eta \tau_{0},\;\theta^{\prime }=\frac{\gamma \left(T-T_{0}\right)}{2\mu +\lambda },$$

$$\sigma_{ij}^{\prime }=\frac{\sigma_{ij}}{\mu +\alpha_{p}},\;\mu_{ij}^{\prime }=\frac{\alpha_{p}\mu_{ij}}{v\eta \left(\mu +\alpha_{p}\right)\left(v_{p}+\beta_{p}\right)},\;\omega_{\varphi }^{\prime }=\frac{\alpha_{p}\omega_{\varphi }}{\mu +\alpha_{p}}.$$

The governing equations are represented in the following forms, where, for the sake of convenience, we have omitted the primes

$$\left(\beta_{1}^{2}-1\right)\nabla [\nabla \;.\;\underline{u}]+\nabla^{2}\underline{u}+2\nabla \;\times \;\underline{\omega }-\beta_{1}^{2}\nabla \theta =\beta_{1}^{2}\underset{-}{\ddot{u}}\;,$$ (16)

$$c_{2}\nabla \;\times \;\underline{u}-c_{1}\underline{\omega }+\left(c_{0}-1\right)\nabla [\nabla \;.\;\underline{\omega }]+\nabla^{2}\underline{\omega }=c_{3}\underset{-}{\ddot{\omega }},$$ (17)

$$\left(\frac{\partial }{\partial t}+\tau_{0}\frac{\partial^{2}}{\partial t^{2}}\right)(\theta +\varepsilon e)=\nabla^{2}\theta .$$ (18)

$$\sigma_{rr}=\left(1+\delta_{1}\right)\;\frac{\partial u_{r}}{\partial r}+\left(\beta_{1}^{2}-1-\delta_{1}\right)e-\beta_{1}^{2}\theta$$ (19)

$${\bar{\sigma }}_{\varphi \varphi }=\beta_{1}^{2}e-\left(1+\delta_{1}\right)\;\frac{\partial u_{r}}{\partial r}-\beta_{1}^{2}\theta$$ (20)

$$\sigma_{r\varphi }=\frac{\partial u_{\varphi }}{\partial z}+\delta_{1}\left(\frac{1}{r}\;\frac{\partial u_{r}}{\partial \varphi }-\frac{u_{\varphi }}{r}\right)-2\omega_{z}$$ (21)

$$\sigma_{\varphi r}=\delta_{1}\;\frac{\partial u_{\varphi }}{\partial r}+\left(\frac{1}{r}\;\frac{\partial u_{r}}{\partial \varphi }-\frac{u_{\varphi }}{r}\right)+2\omega_{z}$$ (22)

$$\mu_{rz}=\frac{\partial \omega_{z}}{\partial r}$$ (23)

$$\mu_{zr}=\delta_{2}\frac{\partial \omega_{z}}{\partial r},$$ (24)

$$\mu_{\varphi z}=\frac{1}{r}\;\frac{\partial \omega_{z}}{\partial \varphi },$$ (25)

$$\mu_{z\varphi }=\frac{\delta_{2}}{r}\;\frac{\partial \omega_{z}}{\partial \varphi }$$ (26)

Where

$$\eta =\rho c_{E}\;/\;k,\;v^{2}=(2\mu +\lambda )\;/\;\rho ,$$

$$\beta_{1}^{2}=\frac{\lambda +2\mu }{\mu +\alpha_{p}},\;c_{0}=\frac{\varepsilon_{p}+2v_{p}}{v_{p}+\beta_{p}},\;c_{1}=\frac{4\alpha_{p}}{v^{2}\eta^{2}\left(v_{p}+\beta_{p}\right)},\;c_{2}=\frac{\alpha_{p}^{2}}{v^{2}\eta^{2}\left(\mu +\alpha_{p}\right)\left(v_{p}+\beta_{p}\right)},$$

$$c_{3}=\frac{J(\lambda +2\mu )}{v_{p}+\beta_{p}},\;\varepsilon =\frac{\gamma^{2}T_{0}}{\rho c_{E}(\lambda +2\mu )},\;\delta_{1}=\frac{\mu -\alpha_{p}}{\mu +\alpha_{p}},\;\delta_{2}=\frac{v_{p}-\beta_{p}}{v_{p}+\beta_{p}}.$$

*v* is the velocity at which isothermal longitudinal elastic waves propagate.

## 3. Solution in the transformed domain

Applying Laplace transform which is defined by the formula [39]

$$\bar{f}(r,\;s)=\int_{0}^{\infty }f(r,t)e^{-st}dt$$

Where, *f*(*r*, *t*) is an arbitrary function

Appling Laplace transform for Eqs (16)–(18) we get:

$$\nabla^{2}\bar{u}+\left(\beta_{1}^{2}-1\right)\nabla [\nabla \;.\;\bar{u}]-\beta_{1}^{2}\nabla \bar{\theta }+2\nabla \;\times \;\bar{w}=\beta_{1}^{2}s^{2}\bar{u},$$ (27)

$$\nabla^{2}\bar{w}+c_{2}\nabla \;\times \;\bar{u}+\left(c_{0}-1\right)\nabla [\nabla \;.\;\bar{w}]=\left(c_{3}s^{2}+c_{1}\right)\bar{w},$$ (28)

$$\left(s+\tau_{0}s^{2}\right)(\bar{\theta }+\varepsilon \bar{e})=\nabla^{2}\bar{\theta }.$$ (29)

Making use of the relations

*∇*^2^ = grad(div)–curl curl,

Eqs, (27) and (28) take the forms

$$\beta_{1}^{2}\nabla [\nabla \;.\;\bar{u}]-\beta_{1}^{2}\nabla \bar{\theta }-\nabla \;\times \;\nabla \;\times \;\bar{u}+2\nabla \;\times \;\bar{w}=\beta_{1}^{2}s^{2}\bar{u}.$$ (30)

$$c_{2}\nabla \;\times \;\bar{u}-\nabla \;\times \;\nabla \;\times \;\bar{w}+c_{0}\nabla [\nabla \;.\;\bar{w}]=\left(c_{3}s^{2}+c_{1}\right)\bar{w}.$$ (31)

The divergence operator is applied for Eq (30), we obtain

$$\nabla^{2}\bar{\theta }=\left(\nabla^{2}-s^{2}\right)\bar{e}.$$ (32)

Eliminating $\bar{e}$ between Eqs (29), and (32), we get

$$\left[\nabla^{4}-\left\{s^{2}+(\varepsilon +1)s\left(\tau_{0}s+1\right)\right\}\nabla^{2}+s^{3}\left(\tau_{0}s+1\right)\right]\bar{\theta }=0.$$ (33)

Similarly, we can show that $\bar{e}$ satisfies the equation

$$\left[\nabla^{4}-\left\{s^{2}+(\varepsilon +1)\left(s+\tau_{0}s^{2}\right)\right\}\nabla^{2}+s^{3}\left(\tau_{0}s+1\right)\right]\bar{e}=0.$$ (34)

The factorized form of Eq (34) will take the following form:

$$\left(\nabla^{2}-k_{1}^{2}\right)\left(\nabla^{2}-k_{2}^{2}\right)\bar{\theta }=0,$$ (35)

where ±*k*_1_ and ±*k*_2_ are the roots of Eq (34)

$$k^{4}-\left[s^{2}+(\varepsilon +1)\left(s+\tau_{0}s^{2}\right)\right]k^{2}+s^{3}\left(\tau_{0}s+1\right)=0,$$ (36)

The solution of Eq (36) can be given as

$$\bar{\theta }={\bar{\theta }}_{1}+{\bar{\theta }}_{2}$$ (37)

where,

$$\left(\nabla^{2}-k_{i}^{2}\right){\bar{\theta }}_{i}=0,\;i=1,\;2.$$ (38)

But

$$\frac{\partial^{2}{\bar{\theta }}_{i}}{\partial r^{2}}+\frac{1}{r}\;\frac{\partial {\bar{\theta }}_{i}}{\partial r}+\frac{1}{r^{2}}\;\frac{\partial^{2}{\bar{\theta }}_{i}}{\partial \varphi^{2}}-k_{i}^{2}{\bar{\theta }}_{i}=0$$ (39)

$$\;\text{Let}\;{\bar{\theta }}_{i}=R(r)\;.\;\Phi (\varphi )\text{,}\;\;\text{then}$$ (40)

$$\frac{r^{2}}{R}\;\frac{d^{2}R}{dr^{2}}+\frac{r}{R}\;\frac{dR}{dr}+\frac{1}{\Phi }\;\frac{d^{2}\Phi }{d\varphi^{2}}-k_{i}^{2}r^{2}=0$$ (41)

We thus get:

$$\frac{d^{2}\Phi }{d\varphi^{2}}=constant\;$$

$$\frac{r^{2}}{R}\;\frac{d^{2}R}{dr^{2}}+\frac{r}{R}\;\frac{dR}{dr}-k_{i}^{2}r^{2}=-\;constant\;$$ (42)

Since *Φ* is periodic with a period of 2*π*, therefore

$$\;constant\;=-\;n^{2},\;n=0,\;1,\;2,\;\ldots$$ (43)

$$R(r)=A_{n1}I_{n}(kr)$$ (44)

$$\Phi (\varphi )=A_{n2}\cos\;n\varphi +A_{n3}sin\;n\varphi$$ (45)

For simplicity, we shall take θ as an even function of φ, thus

$$\bar{\theta }=\sum_{n=0}^{\infty }\sum_{i=1}^{2}\left(k_{i}^{2}-s^{2}\right){\text{I}}_{\text{n}}\left(k_{i}r\right)A_{ni}\cos\;n\varphi$$ (46)

Similarly, the solution of $\bar{e}$ which is compatible with Eq (32) is:

$$\bar{e}=\sum_{n=0}^{\infty }\sum_{i=1}^{2}k_{i}^{2}\;{\text{I}}_{\text{n}}\left(k_{i}r\right)A_{ni}\cos\;n\varphi$$ (47)

Eqs (27) and (28) take the following form:

$$\left(\beta_{1}^{2}-1\right)\nabla [\nabla \;.\;\bar{u}]+\nabla^{2}\bar{u}+2\nabla \;\times \;\bar{w}-\beta_{1}^{2}\nabla \bar{\theta }=\beta_{1}^{2}s^{2}\bar{u},$$ (48)

$$c_{2}\nabla \;\times \;\bar{u}-c_{1}\bar{w}+\left(c_{0}-1\right)\nabla [\nabla \;.\;\bar{w}]+\nabla^{2}\bar{w}=c_{3}s^{2}\bar{w},$$ (49)

Components of Eq (48) are:

$$\beta_{1}^{2}\frac{\partial \bar{e}}{\partial r}-\frac{1}{r^{2}}\;\frac{\partial }{\partial \varphi }\left[\frac{\partial \left(r{\bar{u}}_{\varphi }\right)}{\partial r}-\frac{\partial {\bar{u}}_{r}}{\partial \varphi }\right]+\frac{2}{r}\;\frac{\partial {\bar{w}}_{z}}{\partial \varphi }-\beta_{1}^{2}\frac{\partial \bar{\theta }}{\partial r}=\beta_{1}^{2}s^{2}{\bar{u}}_{r}$$ (50)

$$\beta_{1}^{2}\;\frac{1}{r}\;\frac{\partial \bar{e}}{\partial \varphi }+\frac{\partial }{\partial r}\left[\frac{1}{r}\left(\frac{\partial \left(r{\bar{u}}_{\varphi }\right)}{\partial r}-\frac{\partial {\bar{u}}_{r}}{\partial \varphi }\right)\right]-2\frac{\partial {\bar{w}}_{Z}}{\partial r}-\beta_{1}^{2}\;\frac{1}{r}\;\frac{\partial \bar{\theta }}{\partial \varphi }=\beta_{1}^{2}s^{2}{\bar{u}}_{\varphi }$$ (51)

Eq (49) has only one component, namely

$$\nabla^{2}{\bar{w}}_{z}-\left(c_{1}+c_{3}s^{2}\right){\bar{w}}_{z}=\frac{-c_{2}}{r}\left[\frac{\partial \left(r{\bar{u}}_{\varphi }\right)}{\partial r}-\frac{\partial {\bar{u}}_{r}}{\partial \varphi }\right]$$ (52)

$$\;\text{Let}\;\bar{g}=\frac{\partial \left(r{\bar{u}}_{\varphi }\right)}{\partial r}-\frac{\partial {\bar{u}}_{r}}{\partial \varphi }$$

Then, we have from Eqs (50), (51) and (52), we obtain

$$\beta_{1}^{2}\frac{\partial \bar{e}}{\partial r}-\frac{1}{r^{2}}\;\frac{\partial \bar{g}}{\partial \varphi }+\frac{2}{r}\frac{\partial {\bar{w}}_{Z}}{\partial \varphi }-\beta_{1}^{2}\frac{\partial \bar{\theta }}{\partial r}=\beta_{1}^{2}s^{2}{\bar{u}}_{r}$$ (53)

$$\beta_{1}^{2}\frac{\partial \bar{e}}{\partial \varphi }+r\frac{\partial }{\partial r}\left(\frac{\bar{g}}{r}\right)-2r\frac{\partial {\bar{w}}_{z}}{\partial r}-\beta_{1}^{2}\frac{\partial \bar{\theta }}{\partial \varphi }=\beta_{1}^{2}s^{2}r{\bar{u}}_{\varphi }$$ (54)

$$\nabla^{2}{\bar{w}}_{z}-\left(c_{1}+c_{3}s^{2}\right){\bar{w}}_{z}=\frac{-c_{2}}{r}\bar{g}$$ (55)

Differentiating Eq (53) with respect to *φ* and Eq (54) with respect to *r* and subtracting, we get

$$\frac{\partial }{\partial r}\left[r\frac{\partial }{\partial r}\left(\frac{\bar{g}}{r}\right)\right]+\frac{1}{r^{2}}\;\frac{\partial^{2}\bar{g}}{\partial \varphi^{2}}-2r\nabla^{2}{\bar{w}}_{z}-\beta_{1}^{2}s^{2}\bar{g}=0$$ (56)

Letting, $G=\frac{g}{r}$ Eq (56) becomes,

$$\left(\nabla^{2}-\beta_{1}^{2}s^{2}\right)\bar{G}=2\nabla^{2}{\bar{w}}_{z}$$ (57)

From Eq (55)

$$\left(\left(\nabla^{2}-\left(c_{1}+c_{3}s^{2}\right)\right){\bar{w}}_{z}\right)=-c_{2}\bar{G}$$ (58)

Multiplying Eq (57) by *c*_2_ and Eq (58) by $\left(\nabla^{2}-\beta_{1}^{2}s^{2}r\right)$ and subtracting, we obtain

$$\left[\nabla^{4}-\left(s^{2}\beta_{1}^{2}+\left(c_{3}s^{2}+c_{1}\right)-2c_{2}\right)\nabla^{2}+s^{2}\beta_{1}^{2}\left(c_{3}s^{2}+c_{1}\right)\right]{\bar{w}}_{z}=0$$ (59)

Eq (59) can be factorized as

$$\left(\nabla^{2}-g_{1}^{2}\right)\left(\nabla^{2}-g_{2}^{2}\right){\bar{w}}_{z}=0$$ (60)

where, $g_{1}^{2}$ and $g_{2}^{2}$ are the roots of Eq (59)

As before, we have the solution

$${\bar{w}}_{z}=\sum_{i=1}^{2}{\bar{w}}_{zi}$$ (61)

$$\;\text{where,}\;{\bar{w}}_{zi}\;\text{is}\;\text{the}\;\text{solution}\;\text{of}\;\left(\nabla^{2}-g_{i}^{2}\right){\bar{w}}_{zi}=0$$ (62)

$${\bar{w}}_{z}=\sum_{n=0}^{\infty }\sum_{i=1}^{2}B_{ni}I_{n}\left(g_{i}r\right)\;\text{sin}\;n\varphi$$ (63)

Similarly

$$\bar{g}=r\bar{G}=r\;\sum_{n=0}^{\infty }\sum_{i=1}^{2}\frac{2g_{i}^{2}}{\left(g_{i}^{2}-\beta_{1}^{2}s^{2}\right)}B_{ni}I_{n}\left(g_{i}r\right)sin\;n\varphi$$ (64)

Substituting from Eqs (46), (47), (64) and (64) into Eqs (53) and (54), we get

$$\begin{array}{l} {\bar{u}}_{r}=\sum_{n=0}^{\infty }\sum_{i=1}^{2}A_{ni}\left[\frac{n}{r}I_{n}\left(k_{i}r\right)+k_{i}I_{n+1}\left(k_{i}r\right)\right]\;\cos\;n\varphi \\ +\frac{1}{\beta_{1}^{2}s^{2}r}\sum_{n=0}^{\infty }\sum_{i=1}^{2}\left[\frac{c_{2}n}{\left(g_{i}^{2}-\beta_{1}^{2}s^{2}\right)}+2n\right]B_{ni}I_{n}\left(g_{i}r\right)\;\cos\;n\varphi \end{array}$$ (65)

$$\begin{array}{c} {\bar{u}}_{\varphi }=\frac{-1}{\beta_{1}^{2}s^{2}r}\left[\beta_{1}^{2}s^{2}\sum_{n=0}^{\infty }\;\sum_{i=1}^{2}nI_{n}\left(k_{i}r\right)A_{ni}sin\;n\varphi \right]\\ +\;r\sum_{n=0}^{\infty }\;\sum_{i=1}^{2}\left[\frac{c_{2}}{\left(g_{i}^{2}-\beta_{1}^{2}s^{2}\right)}+2\right]B_{ni}\left(g_{i}I_{n+1}\left(g_{i}r\right)+\frac{n}{r}I_{n}\left(g_{i}r\right)\right)sin\;n\varphi \end{array}$$ (66)

Finally, substituting Eqs (46), (47), (63), (65), and (66) to the Laplace transform of Eqs (19)–(26), to get the Laplace transform of stress components:

$$\begin{array}{c} {\bar{\sigma }}_{rr}=\sum_{n=0}^{\infty }\;\sum_{i=1}^{2}\left(\left(1+\delta_{1}\right)\left(\frac{n^{2}}{r^{2}}-\frac{n}{r^{2}}\right)+\beta_{1}^{2}s^{2}\right)A_{ni}I_{n}\left(k_{i}r\right)cos\;n\varphi \\ +\;\frac{1}{\beta_{1}^{2}s^{2}r^{2}}\sum_{n=0}^{\infty }\;\sum_{i=1}^{2}B_{ni}\left(\frac{c_{2}n}{g_{i}^{2}-\left(c_{1}+c_{3}s^{2}\right)}\right)\left[(n-1)I_{n}\left(g_{i}r\right)+g_{i}rI_{n+1}\left(g_{i}r\right)\right]cos\;n\varphi \end{array}$$ (67)

In order to determine the unknown parameters *A*_*ni*_(*s*) and *B*_*ni*_(*s*), *i* = 1, 2, *n* = 0, 1, 2, … we will proceed by applying the boundary conditions (15)

We can express the function $\bar{f}(\varphi ,\;s)$ as a Fourier cosine series expansion in terms of *φ*, by expanding it as follows:

$$\bar{f}(\varphi ,s)=\sum_{n=0}^{\infty }F_{n}(s)cos\;n\varphi ,$$ (68)

$$\text{Where}\;F_{0}=\frac{1}{\pi }\int_{0}^{\pi }\bar{f}(\varphi ,\;s)d\varphi ,\;F_{n}=\frac{2}{\pi }\int_{0}^{\pi }\bar{f}(\varphi ,s)\;\cos\;n\varphi \;d\varphi$$

Then, the form of the boundary conditions (15) will be:

$$\sum_{n=0}^{\infty }\sum_{i=1}^{2}\left(k_{i}^{2}-s^{2}\right){\text{I}}_{\text{n}}\left(k_{i}r\right)A_{ni}\cos\;n\varphi =\sum_{n=0}^{\infty }F_{n}(s)cos\;n\varphi$$ (69)

$$\begin{array}{c} \sum_{n=0}^{\infty }\;\sum_{i=1}^{2}A_{ni}\left[\frac{n}{r}I_{n}\left(k_{i}r\right)+k_{i}I_{n+1}\left(k_{i}r\right)\right]\cos\;n\varphi \\ +\;\frac{1}{\beta_{1}^{2}s^{2}r}\sum_{n=0}^{\infty }\sum_{i=1}^{2}\left[\frac{c_{2}n}{\left(g_{i}^{2}-\beta_{1}^{2}s^{2}\right)}+2n\right]B_{ni}I_{n}\left(g_{i}r\right)cos\;n\varphi =0 \end{array}$$ (70)

$$\begin{array}{c} \frac{-1}{\beta_{1}^{2}s^{2}r}\left[\beta_{1}^{2}s^{2}\sum_{n=0}^{\infty }\;\sum_{i=1}^{2}nI_{n}\left(k_{i}r\right)A_{ni}sin\;n\varphi \right]\\ +r\;\sum_{n=0}^{\infty }\sum_{i=1}^{2}\left[\frac{c_{2}}{\left(g_{i}^{2}-\beta_{1}^{2}s^{2}\right)}+2\right]B_{ni}\left(g_{i}I_{n+1}\left(g_{i}r\right)+\frac{n}{r}I_{n}\left(g_{i}r\right)\right)sin\;n\varphi \;=\;0 \end{array}$$ (71)

$$\sum_{n=0}^{\infty }\sum_{i=1}^{2}B_{ni}I_{n}\left(g_{i}r\right)\sin\;n\varphi =0$$ (72)

To invert the Laplace transformations in the above Eqs (69)–(72), we used a numerical technique based on Fourier expansion [40].

## 4. Numerical results and discussion

For the purpose of numerical calculations, we shall use a polystyrene material (one of the polymers). The physical parameters values are listed in Table 1 [41].

**Table 1:** Physical parameters.

*k* = 80 W/(m K)	*α*_*t*_ = 8 10^−5^ m^3^kg^-1^	*c*_*E*_ = 3,100 J kg^-1^ K^-1^
*μ* = 1.165 (10)^10^ kg m^-1^ s^-1^	*λ* = 2.262 (10)^10^ kg m^-1^ s^-2^	*ρ* = 1,050 kg m^-3^
*T*_*0*_ = 293 K	*τ*_0_ = 0.02s	*J* = (10)^*−*13^ m^2^
*α*_*p*_ = 2 (10)^9^ kg m^-1^ s^-2^	*β*_*p*_ = (10)^-4^ m kg s^-2^	*v*_*p*_ = 2(10)^*−*4^ m kg s^-2^
$\varphi_{0}=\frac{\pi }{12}$	δ_1_ = -0.263	δ_2_ = 0.33
*c*_1_ = 3.68 (10)^-3^	*c*_2_ = 1.16 (10)^-3^	*c*_3_ = 1.143
$\beta_{1}^{2}=1.45$	*ε* = 0.0104	

From now on, we shall assume that the function *f*(*φ*,*t*) has the following form:

$$f\left(\varphi ,t\right)=H(\varphi_{0}-|\varphi |)H(t),$$

where *H*(.) is the Heaviside unit step function and *φ*_0_ is a constant. Thus, the surface of the cylinder is kept at a constant temperature equal to unity over the sector −*φ*_0_ ≤ φ ≤ φ_0_ and zero everywhere else. The constant *φ*_0_ was taken as $\frac{\pi }{12}$ during computation.

Thus

$$F_{0}=\frac{\varphi_{0}}{\pi s}\;\text{and}\;F_{n}=\frac{2}{n\pi s}\sin\;n\varphi_{0}\;n=1,\;2,\;3\;,\ldots$$

This research presents, for the first time, an innovative direct method for solving the two-dimensional problems of micropolar thermoelasticity. This approach avoids using potential functions with their recognized drawbacks.

Figs 2–4 represent the distribution of the temperature, radial displacement and stresses, respectively with space r, on the diagonal *φ* = 0. Figs 5–9 represent the variation of the functions *θ*, *u*_*r*_, *u*_*φ*_, *σ*_*rr*_ and *w*_*z*_ on the diagonal $\varphi =\frac{\pi }{8}.$

**Fig 2:**
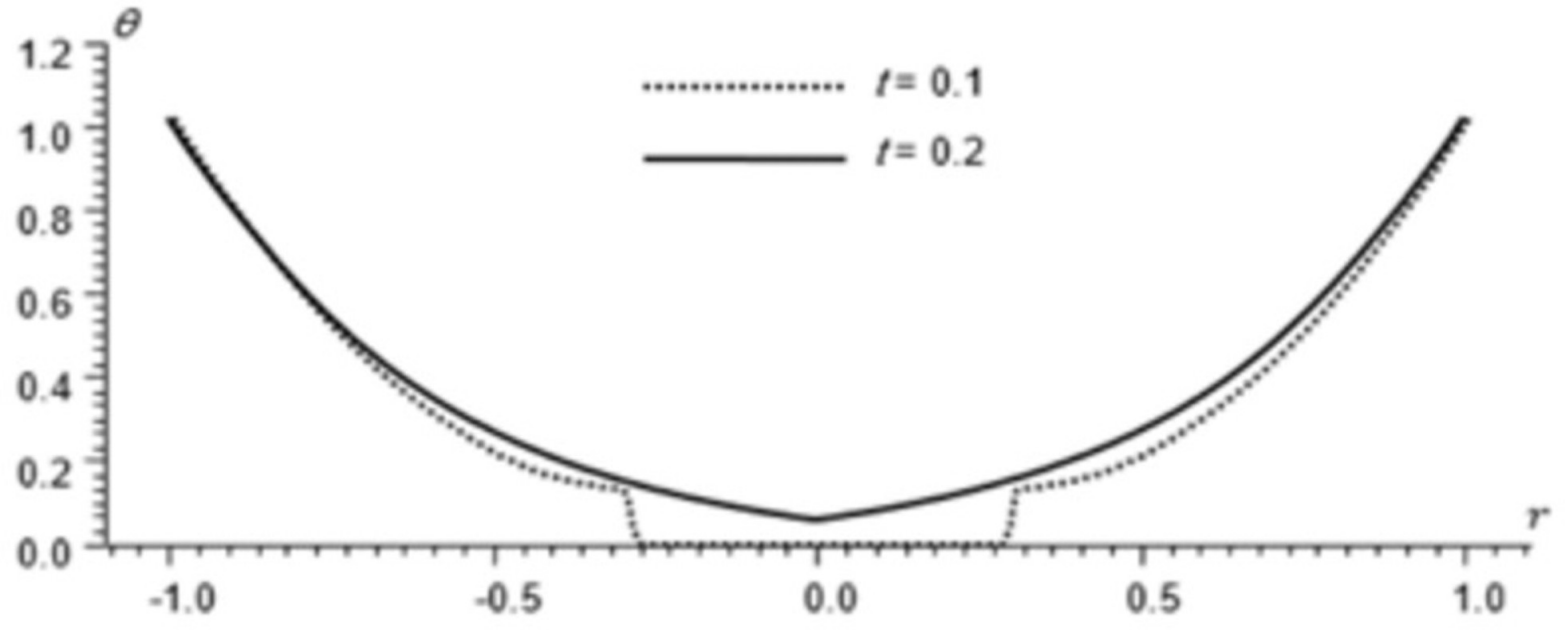
Temperature distribution for different times.

**Fig 3:**
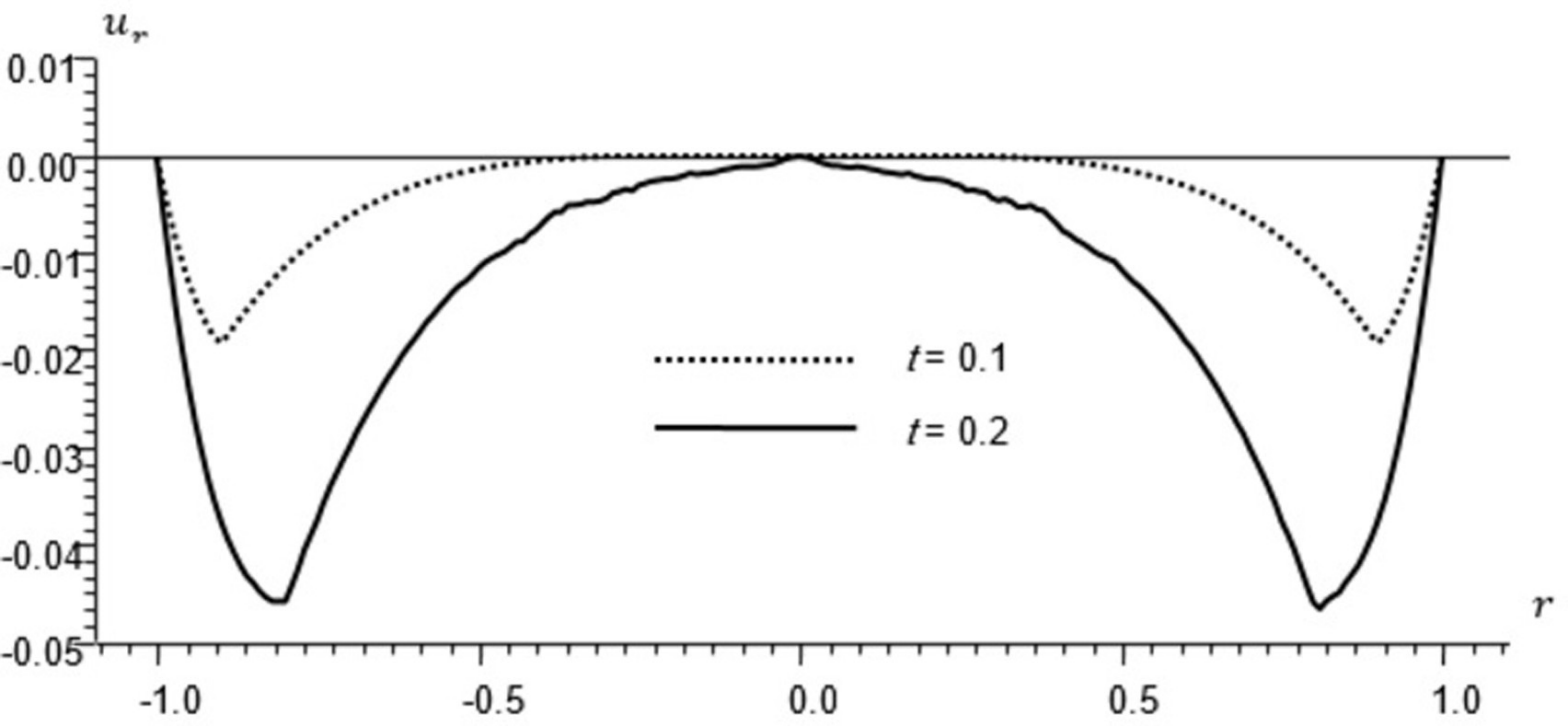
Displacement distribution *u*_*r*_ for different times.

**Fig 4:**
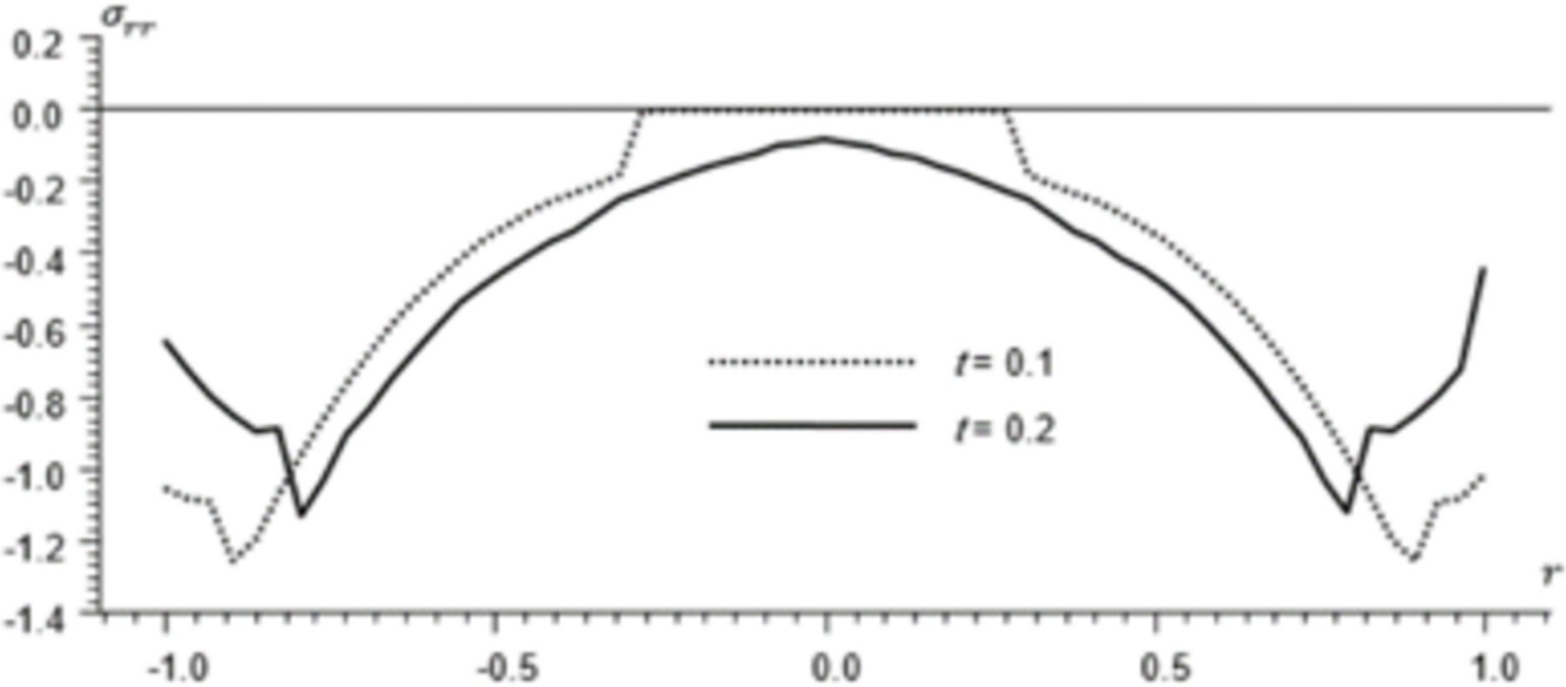
Stresses distribution *σ*_*rr*_ for different times.

**Fig 5:**
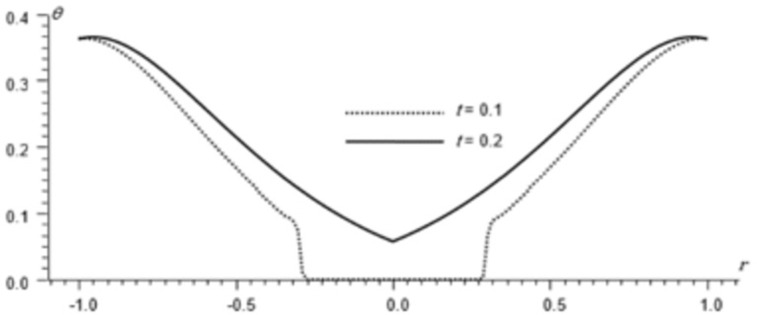
Temperature distribution for different times, $\varphi =\frac{\pi }{8}$.

**Fig 6:**
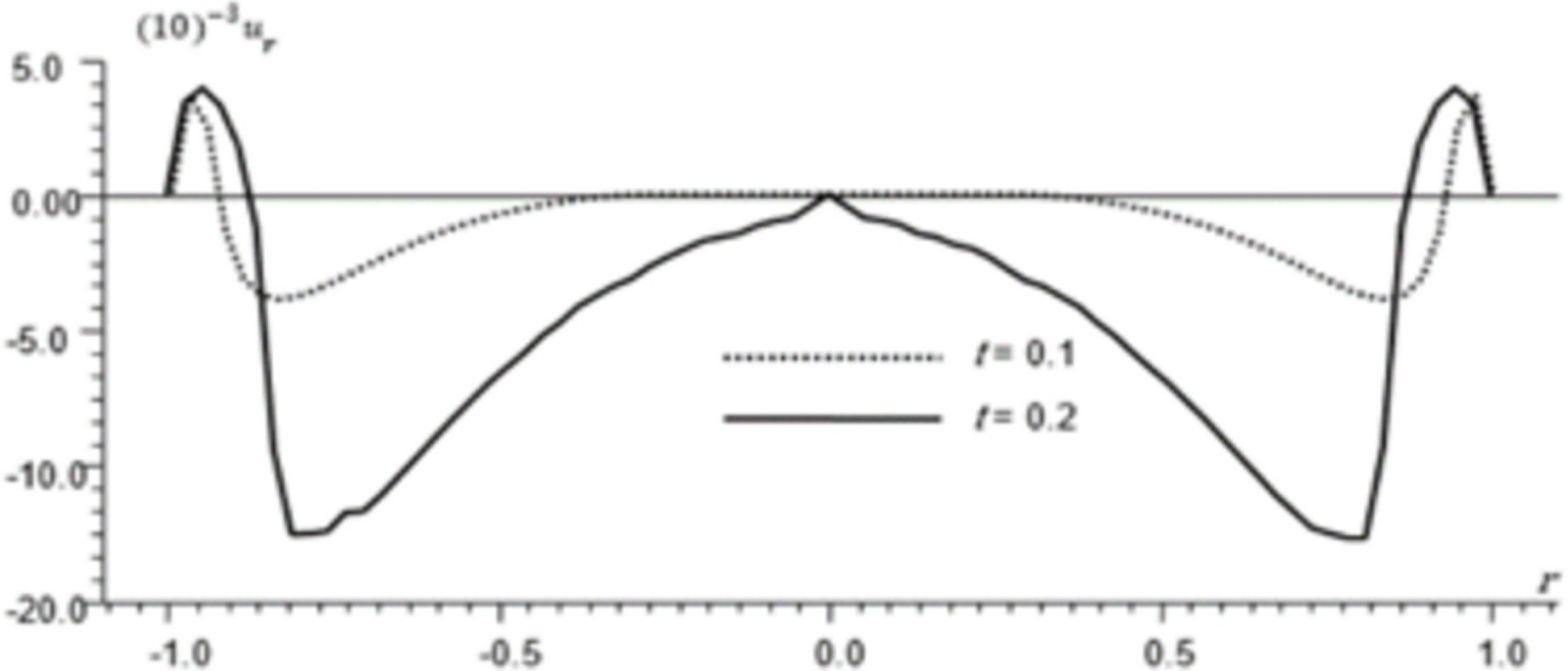
Displacement distribution *u*_*r*_ for different times, $\varphi =\frac{\pi }{8}$.

**Fig 7:**
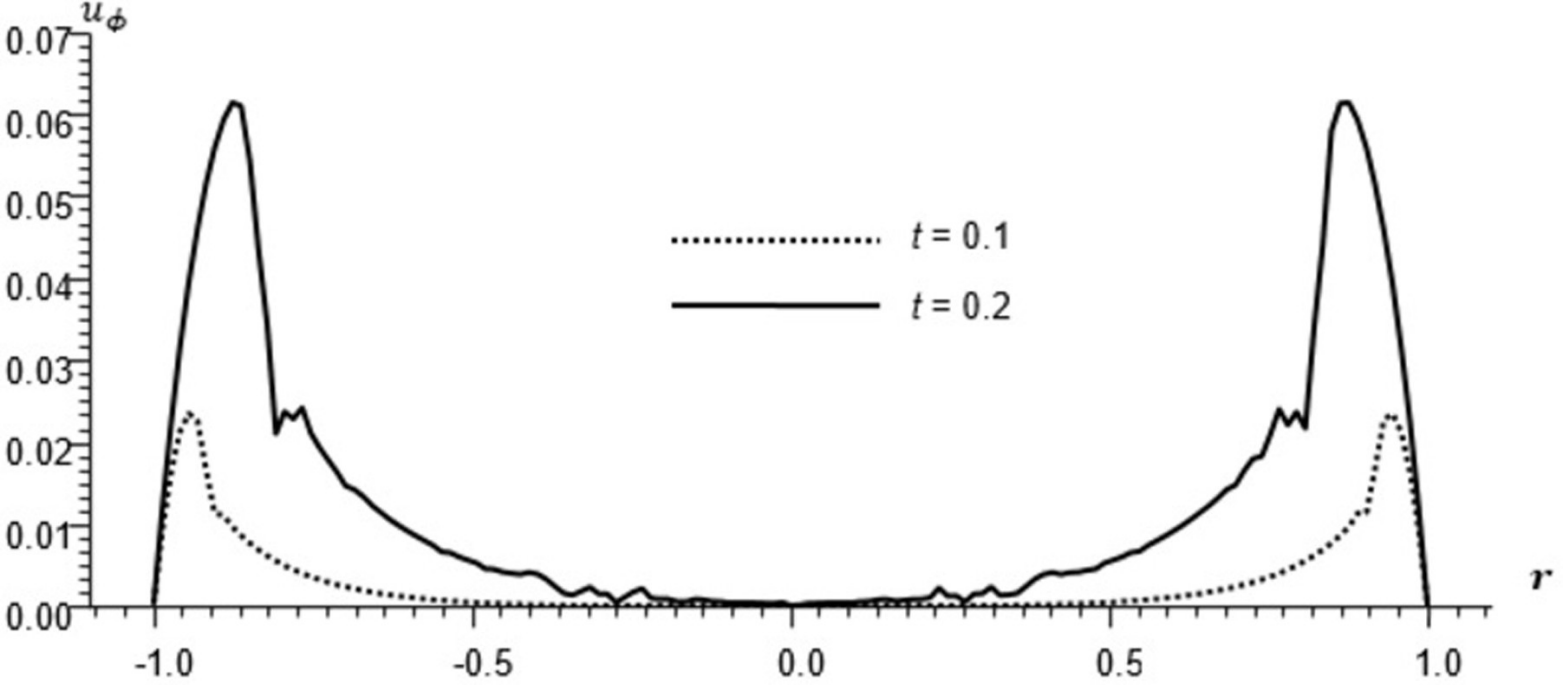
Displacement distribution *u*_*φ*_ for different times, $\varphi =\frac{\pi }{8}$.

**Fig 8:**
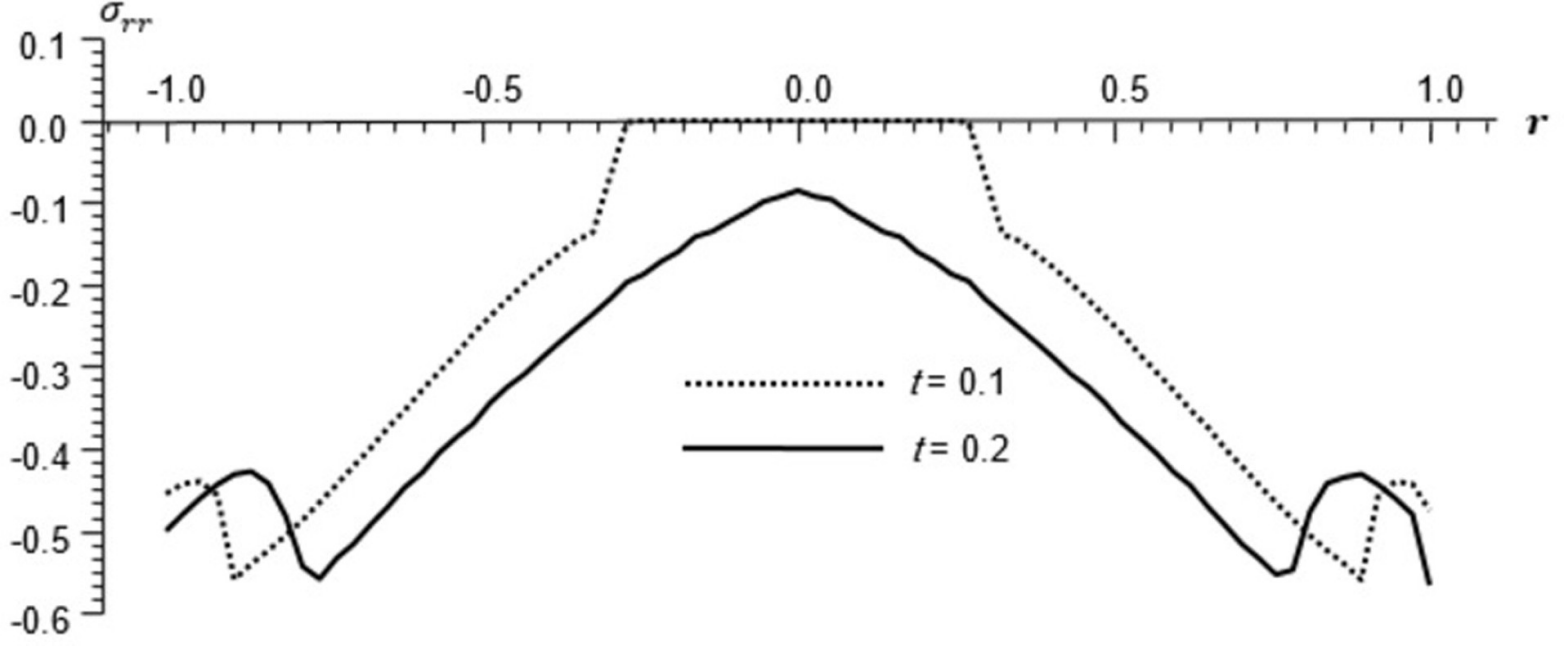
Stresses distribution *σ*_*rr*_ for different times, $\varphi =\frac{\pi }{8}$.

**Fig 9:**
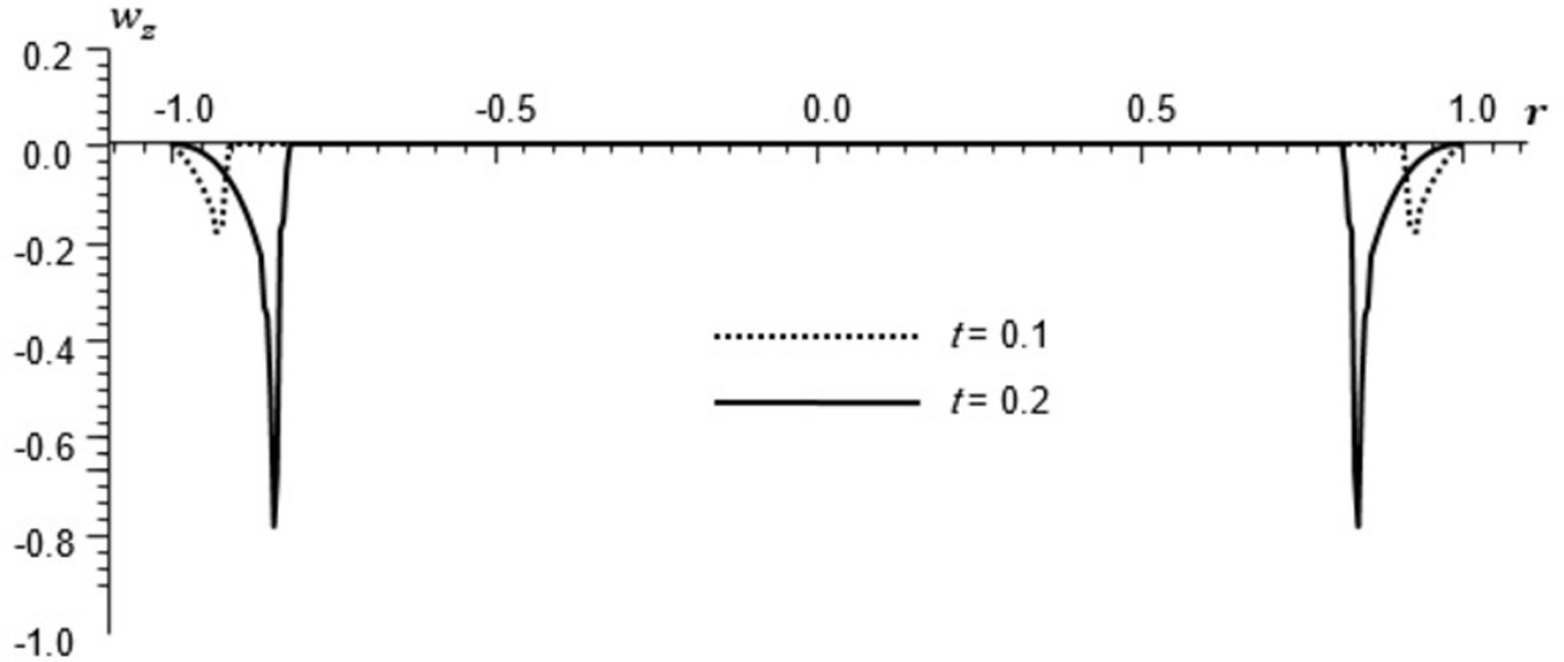
Micro-rotation distribution *w*_*z*_ for different times, $\varphi =\frac{\pi }{8}$.

All the Figs show the fact that the waves in generalized micropolar thermoelasticity travel with finite speeds. The field variables, including temperature, displacement, stresses and micro-rotation, are contingent not just on time (t) and spatial coordinates (r), but also on thermal relaxation time parameters (t1 and t2). It has been noted that, regardless of the values of φ, the relaxation time parameter exerts a substantial influence on field-related variables. In this manuscript, all the variables have been normalized into nondimensional forms. The outcomes pertaining to temperature, displacement, stresses, and microrotation have been obtained by taking t = {0.1, 0.2}. It has been observed that the variation in temperature shown in Figs 2 and 5 increase in magnitude by increasing the relaxation time on the diagonal *φ* = 0 and $\varphi =\frac{\pi }{8}$. Conversely, it has been discovered that the radial displacement demonstrates an inverse proportional relationship with the relaxation time. Figs 3 and 6 represent the distribution of the radial displacement on the diagonal *φ* = 0 and $\varphi =\frac{\pi }{8}$ respectively at different time. The radial displacement decrease in magnitude with the increase of time. On the other hand, the angular displacement distribution, as illustrated in Fig 7, increases as the relaxation time parameter increases. Furthermore, it has been noticed that the radial stress distribution exhibits a similar behavior to that of the radial displacement, as depicted in Figs 4 and 8.

For our problem, we know in advance, that there are four compressional waves (two for each characteristic equation). Two of these waves are similar to their generalized thermoelasticity counterparts. Namely, they are a mainly mechanical wave and a mainly thermal wave. The micropolar impacts are the cause of the other two waves. It was found that they have little effect numerically on the considered functions.

The mechanical wave travels with a velocity of 1.1, approximately, while, the thermal wave travels with a speed of 7.1, approximately.

For the time, *t* = 0.1 the wave front of the mechanical wave as shown in Figs 3 and 4 has crossed a distance of 0.11, indicating a speed of 1.1. The faster thermal wave has crossed a distance of 0.71 from each wall of the cylinder. The location [-0.29, 0.29] has a solution that is identically zero for all functions.

For the time, *t* = 0.2 the waves emanating from both sides have crossed the midpoint filling the whole cylinder. The mechanical wave greatly affects the displacement and stress but has small effect on *θ*. The thermal wave affects both *θ*, *σ*_*rr*_ but has a small effect on the displacement.

From Fig 9 we conclude that one of the waves of the micropolar effects travels with a speed of unity, approximately. The other micro wave has a small impact and cannot be studied numerically. The authors are preparing a manuscript that will evaluate the speeds of these waves analytically in an exact way.

The temperature and stress are discontinuous at the wave front. The first discontinuity in *θ* is very small to observe in the graph. On the other hand, the displacement is continuous for all values of *r* but has discontinuous first derivatives at the wave front.

## 5. Conclusions

1. We have established for the first time a mathematical model that characterizes the interplay between mechanical and thermal influences in micropolar generalized thermoelasticity, employing a novel direct approach.
2. This new approach has been developed to solve problems of asymmetric 2D micropolar generalized thermoelasticity. The main benefit of employing this new approach lies in its avoidance of relying on potential functions. The drawbacks of potential functions are, first, that they are not available always. Secondly, sometimes the solution of the potential functions is divergent while the solution in terms of physical variables is always convergent.
3. The field variables, including temperature changes, displacement distributions, stress variations, and microrotation distributions, are obtained across different relaxation time values in an infinite cylinder with its surface subjected to asymmetric thermal shock. It has been noted that, regardless of the values of φ, the relaxation time parameter exerts a substantial influence on all the distributions.
4. The generalized micropolar thermoelasticity theory for 2D problems implies the existence of four longitudinal waves moving with finite speeds. Three of these waves can be studied numerically while the fourth wave has a small impact and cannot be studied numerically.
5. The components of displacement remain consistently continuous at the wave fronts, whereas all other functions, including temperature, stress components, and micro-rotation, exhibit discontinuity.

## Supporting information

S1 File (TXT)

S2 File (TXT)

S3 File (TXT)

S4 File (TXT)

S5 File (TXT)

S6 File (TXT)

S7 File (TXT)

S8 File (TXT)
